# Consultation-Liaison Psychiatry in Italy: historical development and models of intervention

**DOI:** 10.1192/j.eurpsy.2024.89

**Published:** 2024-08-27

**Authors:** G. Mattei

**Affiliations:** Association for Research in Psychiatry, ARPSY, Castelnuovo Rangone, Italy

## Abstract

**Abstract:**

The birth of Consultation-Liaison (CLP) in Italy was made possible thanks to Act 180/1978, which started a 20-year-process that led to closing asylums and fostered the implementation of small psychiatric units within the general hospitals. In the meantime, Italian CLP grew steadily, fostered also by the enactment of two “Objective Mental Health Care” Plans (1994-1996 and 1998-2000), that led to the implementation of the organizational model of the mental health department (MHD).

As far as psychiatric referrals are concerned, the first Plan states that the MHD covers all territorial and hospital-based activities, in order to assure, among other services, the integration with hospital (with special attention paid to the Emergency Department and to consultation activity in non-psychiatric hospital wards) and general medicine (as well as other sectors, including mother and child health care).

With respect to psychiatric referral, the Second Plan states that “In the 24 months following the entry into force of the Plan, MHDs will adopt guidelines and procedure concerning several issues, including consultation-liaison activity in non-psychiatric hospital wards (which also includes mental health care for “psychiatric” patients hospitalized for non-psychiatric disease in the general hospital) and in Department of Addictions (for alcohol and other substances use disorders. According to the second Plan, Community Mental Health Services (CMHCs) provide, among other performances, CLP activity for general practitioners. Inside general hospitals, psychiatric referrals to non-psychiatric wards are provided by inpatient psychiatric units, when CLP Services are not available.

Since a standard definition of psychiatric consultation is not available, the one provided by the Italian region Emilia-Romagna will be used. The Region includes “consultation” among mental health services provided by CMHCs, and defines it as follows: “Psychiatric or psychological assessment carried out upon request from other [non psychiatric] Departments. The consultation includes both clinical interview with the patient and the medical report for the department that referred the patient.” The ways to identify consultation as one among other types of services provided by the MHD are defined, to count the overall number of consultations and monitor the clinical activity of health professional working in the different branches of the MHD. The following types of consultations are reckoned: consultations requested by general practitioners (who work within the Primary Care Department); consultations requested by the Department of Addiction (a branch of the MHD); and consultations requested by the Department of Child-Adolescent Psychiatry (which, as the previous one, is a branch on the MHD).

**Disclosure of Interest:**

None Declared

